# Dynamic network approach for the modelling of genomic sub-complexes in multi-segmented viruses

**DOI:** 10.1093/nar/gky881

**Published:** 2018-10-09

**Authors:** Kinda AlShaikhahmed, German Leonov, Po-Yu Sung, Richard J Bingham, Reidun Twarock, Polly Roy

**Affiliations:** 1 Faculty of Infectious and Tropical Diseases, London School of Hygiene and Tropical Medicine, Keppel Street, London WC1E 7HT, UK; 2 Departments of Mathematics and Biology & York Cross-disciplinary Centre for Systems Analysis, University of York, York YO10 5GE, UK

## Abstract

Viruses with segmented genomes, including pathogens such as influenza virus, Rotavirus and Bluetongue virus (BTV), face the collective challenge of packaging their genetic material in terms of the correct number and types of segments. Here we develop a novel network approach to predict RNA–RNA interactions between different genomic segments. Experimental data on RNA complex formation in the multi-segmented BTV genome are used to establish proof-of-concept of this technique. In particular, we show that *trans* interactions between segments occur at multiple specific sites, termed segment assortment signals (SASs) that are dispersed across each segment. In order to validate the putative *trans* acting networks, we used various biochemical and molecular techniques which confirmed predictions of the RNA network approach. A combination of mutagenesis and reverse genetics systems revealed that the RNA–RNA interacting sites identified are indeed responsible for segment assortment and complex formation, which are essential criteria for genome packaging. This paves the way for their exploitation as novel types of drug target, either to inhibit assembly, or for designing defective interfering particles containing an incomplete set of genomic segments.

## INTRODUCTION

Viruses are often defined as organisms comprising genetic code protected by a protein container. Whilst this simplification is an adequate description in principle, understanding the details of how viral genomes are packaged into their protective coats, or capsids, represents one of the foremost challenges in virology. This is especially so for viruses with multipartite genomes, as a copy of each segment must be incorporated for the virus to be viable. The mechanism by which this is achieved in competition with the panoply of other nucleic acids present in the infected cell has proved challenging, despite its critical importance to virus replication and survival. Insect-borne Orbiviruses (14 related serogroups; 140 members), a genus in the family *Reoviridae*, have multi-segmented genome. These viruses are transmitted by arthropods (e.g. mosquitoes, gnats and ticks) causing disease in animals and plants, often with a high economic impact on agriculture and animal health. Although Bluetongue virus (BTV, 27 serotypes) and the related epizootic haemorrhagic disease virus (EHDV) of deer, African horse sickness virus (AHSV) are all transmitted by biting midges (gnats, *Culicoides* sp.), BTV is the most common throughout the world including Europe and UK (1–3). Consequently, BTV has been the subject of extensive studies as a model system (4,5).

BTV is structurally highly complex with a genome of 10 double-stranded RNA segments enclosed by a multi-layered protein capsid. It shares a virus family relationship with several other veterinary and medically important viruses (e.g. Rotaviruses). The BTV RNA segments vary in sizes (3.95–0.8 kb) and sequence, but each shares common short 5′ and 3′ untranslated regions (UTRs) of variable length, including highly conserved hexanucleotides at both ends (6). Recent data suggested that RNA segments are likely to be responsible for formation of an RNA complex via intersegment interactions prior to packaging (7–12).

In order to elucidate the mechanism of RNA segment assortment, we developed the first dynamic network approach for the modelling of segment association in multi-segmented viruses. Our analysis focuses on all stages of RNA segment assembly, reassessing interaction probabilities at each stage of complex formation, up to a complex formed from the five shortest segments of BTV, i.e. S10 to S6, which were computationally feasible. Integrating this technique iteratively with previously established experimental methods, we identify a set of sequences that are vital for the formation of this intermediate complex and thus virus infectivity. In particular, network analysis predicts the likely *trans* interactions between segments, given the probabilities of *cis* interactions for each individual one. Predicted interaction sites were validated by mutagenesis, electrophoresis mobility shift assays (EMSAs) and virus recovery using reverse genetics. Our data describe the essential drivers of segment assortment in BTV as multiple sites dispersed along the full length of the segments but not localized in a single site of each segments as described previously (11). Our integrated interdisciplinary approach provides unique methods and insights into the formation of RNA complexes that can also be applied to other segmented RNA viruses.

## MATERIALS AND METHODS

### Identification of *trans* interactions

The list of all possible candidates for *trans* interactions of genomic fragments in different segments with average (*cis*) base pairing probabilities of <75% was compiled using RNAplex (13). RNAplex enables identification of possible hybridization sites between RNAs, in particular short, highly stable interactions, via a dynamic programming algorithm. The algorithm focuses on loop types that are stacked pairs, bulge loops or interior loops, with free energy contributions from stacked pairs and small interior loops determined via Turner energy parameters. For bulge loops of length 1 the stacking energy of the two pairs closing the loop is taken into account together with a sequence independent penalty, whilst larger bulge-loops are assigned a length-dependent penalty. A detailed description of the algorithm can be found in (13), together with a demonstration that it performs efficiently and reliably on experimentally determined miRNA–mRNA contacts. The code is publicly available via http://www.bioinf.uni-leipzig.de/Software/RNAplex/. Interactions were ranked by free energy of formation, and mutually exclusive options pruned, giving preference to those with lower energy and larger contiguous stretches of base-pairings, and thus higher probability of formation. Any such interactions with free energy of formation of –15 kcal/mol or lower are retained for network analysis.

### Network analysis using Gephi

Generation of input files for Gephi (14) was automatized for the table of *trans* interactions. A node was associated with the centre of every *trans* interaction, as well as *cis* interactions if their binding partners are located on either side of a *trans* interaction along the linear genomic sequence. Networks were displayed using force atlas 2, using the prevent overlap and approximate repulsion functions and an edge weight influence of 0.5.

### Plasmids, mutagenesis and RNA transcripts synthesis

T7 transcripts were generated from exact cDNA copies of the BTV-1 genome using methods described previously (15). All mutations of S6 and S10 were generated by site-directed mutagenesis and joined using the Gibson assembly kit. Sequences of mutations and primers are listed in Supplementary Figure S5.

### *In vitro* transcription and electrophoretic mobility shift assay (EMSA)

As described previously (9), T7 DNA constructs of S6-S10 were mixed in equal molar quantities and RNAs were simultaneously co-transcripted. The RNA samples were analysed with agarose gel.

### Reverse genetics

Following methods described previously (9), S6 or S10 containing mutations in #20 regions that do not change amino acids (S6 #20 [nt985–1005]: 5′-ATCGGTAGCCCAGGTAGCGTG-3′; S10 #20 [nt21–41]: 5′-TGTTGAGTGGCTTAATACAGA-3′), together with other nine BTV genome segments were used to transfect BSR cells. CPE was monitored after 3 days.

### qRT-PCR

Following methods described previously (8), retarded bands were excised and RNA extracted; RT and qPCR were then performed using specific BTV S6–S10 primers.

## RESULTS

### Prediction of interaction sites during BTV genomic RNA complex formation

**Figure 1.**
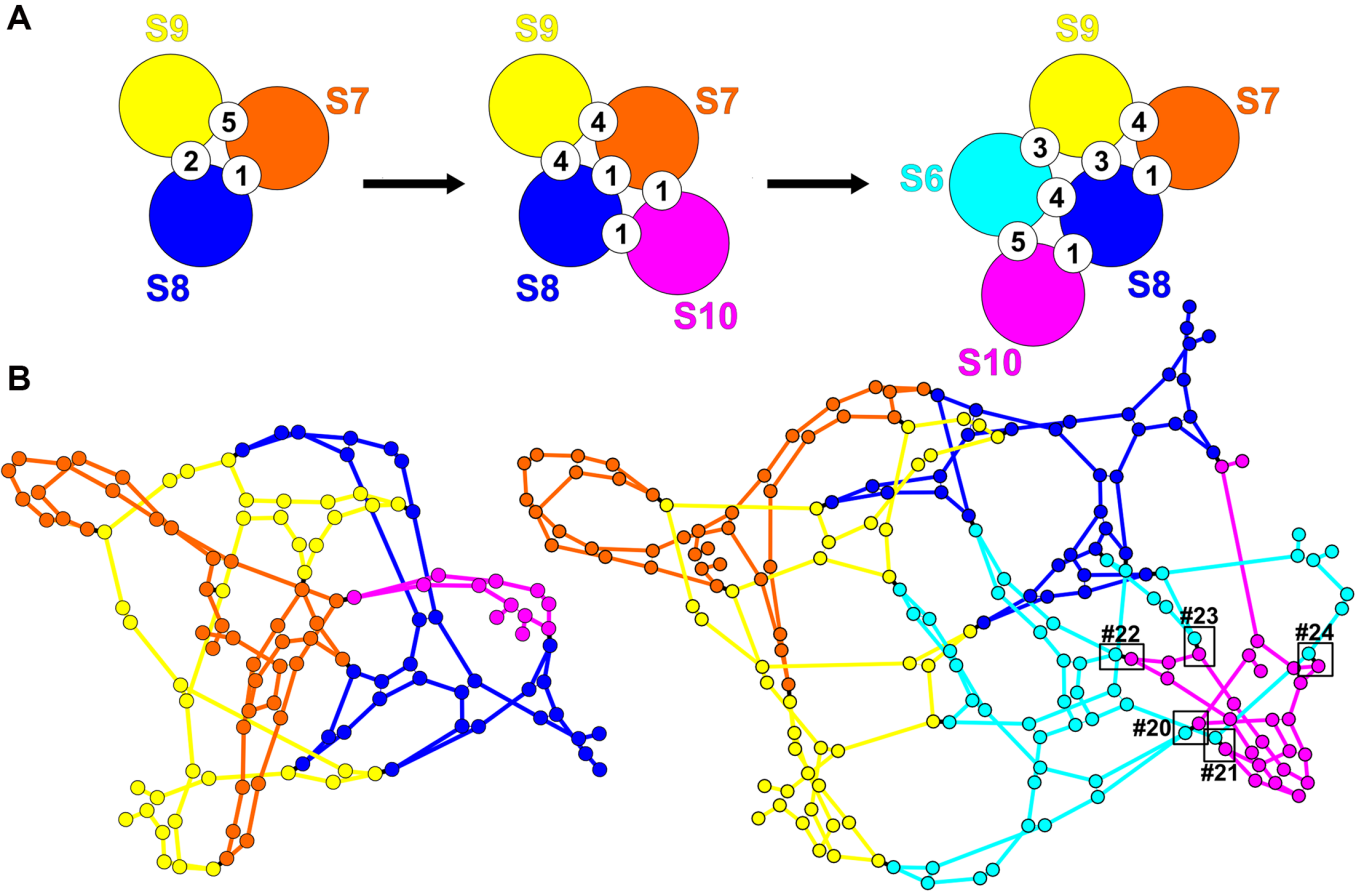
The predicted network of *trans* interactions. (**A**) Cartoon illustrating the changing distributions of *trans* interactions during transition from Stage 1 to Stage 3. (**B**) The interaction networks at Stages 2 and 3 are illustrated in Gephi; individual segments are colour-coded as in (A), and nodes represent positions of *cis* or *trans* interactions.

The 10 individual RNA segments of BTV are recruited and packaged in sequential order, mediated by specific multi-site RNA interactions (8,9). As *in vitro* cell-free assembly (CFA) assays and beads binding RNA–RNA interaction assays suggest that smaller segments are more critical for BTV genome assembly (8,9,16), we focus here on assembly of the subcomplex formed by the five smallest segments S10 to S6. In order to determine putative interactions between these segments, we start by identifying the most likely self-interactions for each individual segment. Based on an ensemble of 1000 sample folds obtained via S-fold (17), we identify all nucleotides that are base-paired in at least 75% of the samples, as these are more likely to be involved in *cis* than in *trans* interactions. This threshold was chosen as it is consistent with a previously reported *trans*-RNA-interaction region on S10, namely the S10.2 antisense oligonucleotides (ORN) targeting region (nt 699–737), which is critical for virus replication and RNA complex formation (9). For the complementary regions, i.e. those with less than 75% base-pairing probability, all possible *trans* interactions with a low mean free energy (MFE) of formation (<–15 kcal/mol) and with mostly contiguous base-pairing were identified via RNAplex (see Materials and Methods). Due to the dynamic nature of the interaction network, both steps need to be recomputed iteratively in the growing complex, because interaction probabilities recalibrate across the entire segment once a new interaction has been formed. To identify putative interaction sites between different segments in the complex with incoming segments (or groups of segments), sites of existing *trans* interactions in the complex need to be factored into the sampling of the conformations of RNA segments that already form part of the complex. For this, the predicted *trans* interaction sites are used as constraints on the folds of the interacting segments, i.e. interaction probabilities are sampled based on the understanding that a site already engaged in an interaction is no longer available to interact. This recalibrates all interaction probabilities, and in some cases makes interactions highly favourable that previously only have had a low probability, thus imposing a sequential order on interactions at different stages of complex formation. This dynamic and iterative sampling of the secondary structures of the segments in the growing complex is thus an essential step, and indeed is increasingly important with complex size. It was applied here to identify *trans* interactions at three stages of complex formation: Formation of the initial S7+S8+S9 complex (Stage 1), association of the latter with S10 (Stage 2), followed by association with S6 (Stage 3). The numbers of interactions and the geometries of the Stage 2 and 3 complexes rendered with Gephi (see Materials and Methods) are shown in Figure 1; details of the interactions at all stages, including all 24 interaction sites and their sequences in the S6–S10 complex, are summarized in the Supplementary Material (Supplementary Tables S1 and S2 and Supplementary Figures S1–S3).

Among others, Figure 1A reveals transient contacts that could potentially play a role at earlier stages of complex formation, and that are superseded by alternative contacts once the complex grows.

It also demonstrates the importance of the S6–S10 contacts at Stage 3. Both their number (Figure 1A) and their positions in the complex (boxes in Figure 1B) suggest that they should be vital for complex formation, especially since many of these interactions have a low energy threshold and high probability of interaction.

### A characteristic signature pattern of RNA complexes assembled by S6–S10

**Figure 2.**
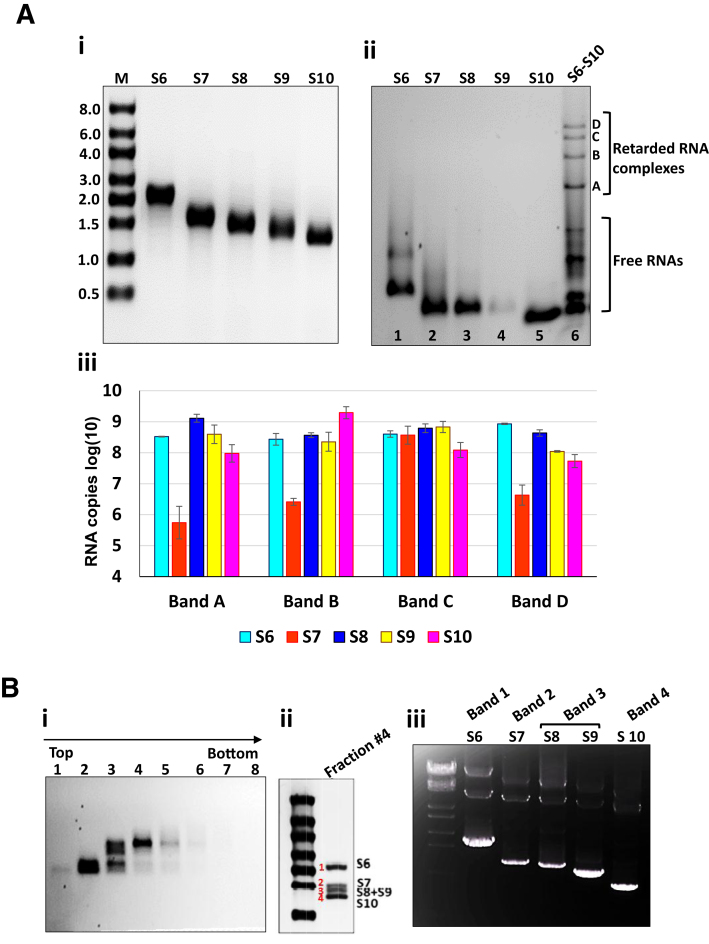
RNA–RNA interactions and complex formation between BTV segments. (**A**) EMSA and qRT-PCR analysis: (**i**) Single T7 transcription of BTV S6-S10 from cDNA. (**ii**) Native agarose gel shows the individual segments (lanes 1–5) or co-transcription of S6-S10 (lane 6). Retarded RNA complexes (bands A-D) and free RNAs are indicated. (**iii**) The quantities of S6–S10 in each retarded band were measured by qRT-PCR. The log (10) quantities are shown in bars, indicating standard deviations from multiple experiments. (**B**) Analysis by Sucrose gradient: **i** Co-transcribed complexes were subjected to a sucrose gradient ultracentrifugation, fractionated, RNA bands identified by EMSA. (**ii**) S6–S10 from fraction #4 are identified by denaturing gel. (**iii**) 1% gel electrophoresis of PCR products of fraction #4 bands extracted from denaturing gel.

To validate the mathematical model of RNA complexes between S6–S10, we used an established *in vitro* RNA–RNA interaction assay (9,12). Each RNA transcript was synthesized individually from T7 plasmids, purified and the molecular size of each was confirmed by denaturing agarose gel electrophoresis (Figure 2A, panel i). Subsequently, equimolar amounts of S6–S10 were co-transcribed and the products were analysed by a native agarose gel electrophoresis mobility shift assay (EMSA) as described previously (9). In addition to the five RNA segments, four retarded RNA bands (designated as A, B, C, D) with slower migration patterns than any individual segment were visualized, indicating that larger RNA complexes were formed (Figure 2A, panel ii). The discrete nature of these four bands was consistently observed in multiple experiments, confirming that these bands are the characteristic signature bands of S6-S10 complexes. There were also several lower bands (indicated as Free RNAs), which consisted of mainly S7–S9, but very little, if any, the other two segments, S6 and S10 (data not shown). Further, a lower mobility band corresponding to S6 dimerization was also visualized in the S6 lane (lane 1). These data suggested further that RNA complexes are indeed formed sequentially.

To determine the composition of RNA complexes visualised as multiple bands in EMSA, qRT-PCR was performed using specific primers to quantify the presence of each RNA segment in each of the four bands (Figure 2A, panel ii, bands A–D). Surprisingly, all five segments were detected in each band, albeit in different quantities (Figure 2A, panel iii). Only band C, the second slowest moving band in the agarose gel, had each segment (S6–S10) in approximately equivalent quantities. In the other three bands, S7 was significantly less; in band A, S7 is ∼1000-fold less than the other segments, while S6 and S9 were comparatively evenly distributed; all bands contained reasonably high level of S10, except band B, which contained >10 folds higher than the other bands.

This is consistent with the predicted interactions (Figure 1A). In particular, S7 and S10 are predicted to make the smallest number of contacts (5 and 6 respectively, as opposed to 12 for S6, 9 for S8, 10 for S9) at Stage 3. Moreover, S7 undergoes the strongest changes in its interactions with other segments, with an interaction between S7 and S9 lost during the transition from Stage 1 to Stage 2, i.e., upon recruitment of S10 to the complex, and S9 now interacting with S8 instead of S7 at that same binding site. Moreover, at Stage 2, S10 forms a transient contact with S7 that is lost again at Stage 3. Indeed, S6 forms new contacts with all segments but S7, with S7 now being the least connected segment in the complex.

To confirm further that all five RNA segments assemble as one complex, the co-transcription reaction mixture was subjected to a 15–65% sucrose gradient ultracentrifugation, followed by fractionation and EMSA analysis (Figure 2B, panel i). When the visible bands of fractions #2-#4 were analysed by denaturing gel electrophoresis, only fraction #4 showed the typical profile of RNA complexes as reported previously (9,16) by cell-free assembly assay (Figure 2B, panel ii). To confirm all five segments, S6–S10 were present in the fraction #4, each band from denaturing gel was subsequently extracted and amplified by PCR using specific primers. The products were analysed by gel electrophoresis, which confirmed the presence of all five segments in the complex isolated from gradient (Figure 2B, panel iii).

### Confirmation of specific RNA interaction sites of S6 responsible for RNA complex formation

**Figure 3.**
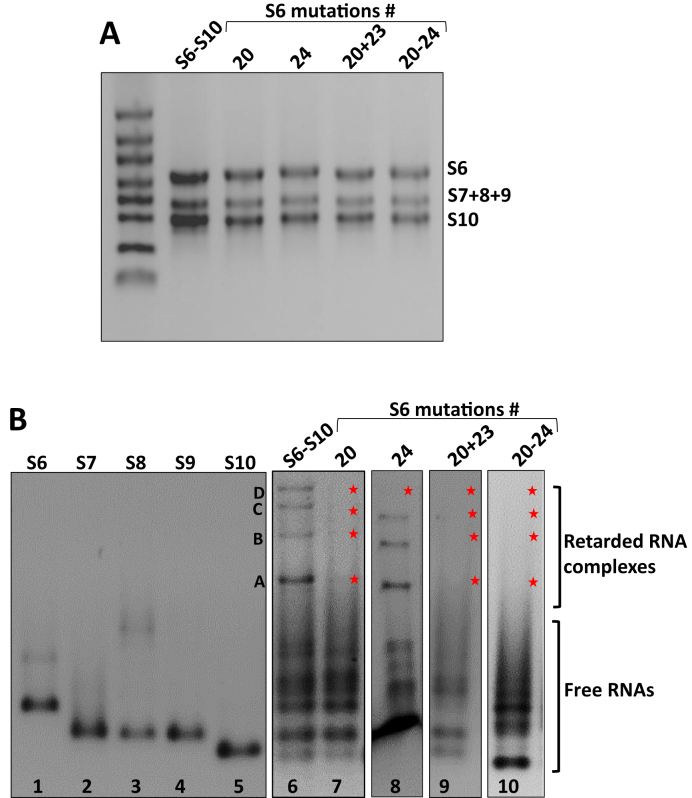
Complex formation of the mutant S6 with wt S7–S10 segments. (**A**) Co-transcribed complexes in denaturing gel show all five transcripts in the correct sizes. (**B**) Native gels of the complexes S7–S10 + S6 (#20) (lane 7), S7–S10 + S6 (#24) (lane 8), S7–S10 + S6 (#20 + #23) (lane 9) and S7–S10 + S6 (#20–#24) (lane 10). RNA complexes are indicated as A–D bands and a star indicates loss of a retarded band. Individual transcripts and co-transcription of wt S6–S10 complexes are used as controls.

In order to investigate the importance of the S6–S10 complex for segments assortment and virus infection, a series of substitution mutations were introduced in S6 and their influences on complex formation determined. Since interaction between S6 and S10 are predicted to be strongest, involving five different sites with high interaction probabilities (Figure 3S; #20-#24), we targeted all these five sites via mutagenesis on an individual basis and in combinations and compare the resulting complexes with those that are characteristic of segment assembly in wild-type (wt) virus. Four mutated constructs were generated, targeting #20 (nt 985–1005), #24 (nt 60–72), a combination of #20 and #23 (nt 1391–1404), and a combination of all five sites #20–#24. Each of these four mutations were substituted by scrambled sequences (Supplementary Figure S5), confirmed by sequencing, and subsequently utilized to co-synthesize ssRNAs by *in vitro* T7 transcription assay. The S6 mutant at position #20, (the predicted strongest interaction site, –27.2 kcal/mol) did not form retarded bands when co-transcribed together with the other four segments (Figure 3B, lane 7), indicating that the mutation at this region destroyed RNA complex formation, consistent with the prediction. When a predicted weaker interaction region, such as #24 (–16.2 kcal/mol) was targeted, only band D disappeared (Figure 3B, lane 8). When both #20 and #23 were mutated in the same construct, all RNA complexes were perturbed (Figure 3B, lane 9). As expected, none of the four bands could be detected when all five sites (#20–#24) were substituted and products analysed (Figure 3B, lane 10). As controls, all the reactions were tested with denaturing agarose gel, confirming that RNA transcription was not influenced by these mutations (Figure 3A).

**Figure 4.**
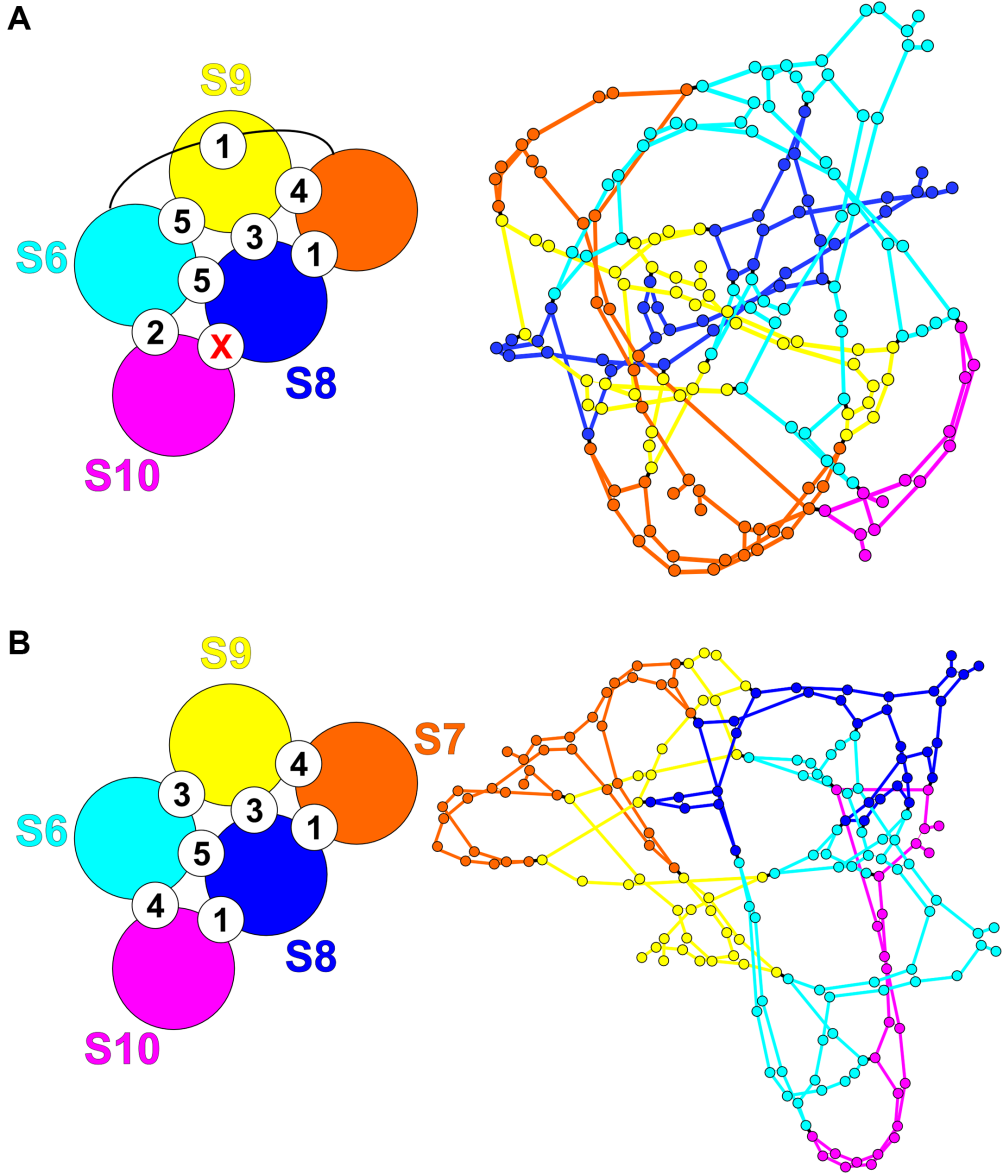
The predicted changes in interactions after multiple mutagenesis of S6 sites. Interactions for (**A**) S7–S10 + S6 (#20–#24) and (**B**) S7–S10 + S6 (#20 + #23).

These observations are consistent with the predicted wt interaction network, and the potential alternative interactions that can occur between S10 and other segments at the sites of the mutated S6–S10 contacts. For example, mutagenesis of #20 frees up S10 to form contacts with S7 at either nts 231–243 (–15.7 kcal/mol) or at nts 479-490 (–16.4 kcal/mol). The interaction network implies that both are unlikely to occur, the former, due to competition with a pre-existing contact (S7-S9), and the latter, due to the distance of the putative interaction sites in the network, consistent with the absence of any complex in Figure 3B, lane 8. Multiple mutations as in the case of S7-S10 + S6 (#20 + #23) and S7–S10 + S6 (#20–#24) in Figure 3b (lanes 6 and 10) can have dramatic impact on network structure due to their roles in *cis* interactions. We therefore recomputed the interaction networks for these cases (Figure 4 and Supplementary Tables S2 and S3). Major changes in geometry and topology of the interaction network with respect to wt (Figure 4A, right) are consistent with the absence of any bands in that case.

**Figure 5.**
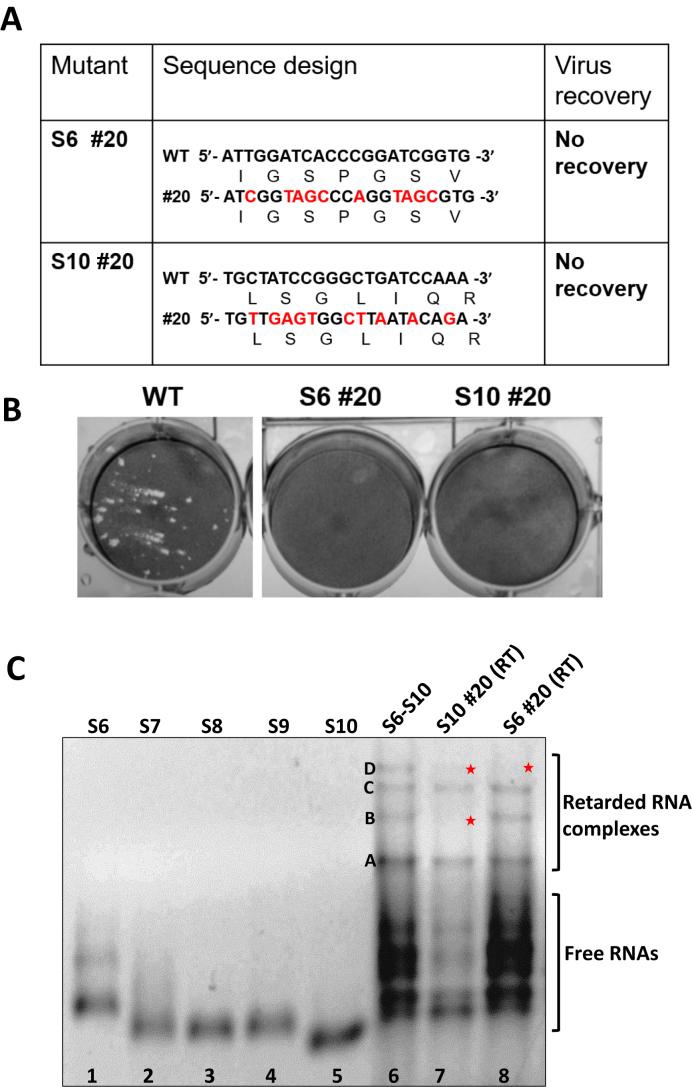
Impact of S6 and S10 mutations on virus recovery. (**A**) Nucleotides in S6 #20 or S10 #20 regions were changed silently, i.e. without changing the encoded amino acids. (**B**) Virus recovery by the RG system is shown by plaque assay. (**C**) Native gel of complex formation of the silent mutations in region #20 of S6 and S10. S7–S9 + S10 (#20) (lane 7) and S7–S10 + S6 (#20) (lane 8).

To confirm that RNA complex formation is essential for virus replication, the predicted highest affinity SAS site on S6 (#20) was mutated in the viral genome using a BTV reverse genetics system. As S6 encodes NS1, an essential viral protein, the silent mutation was designed, i.e. aiming for maximum nucleotide changes without altering the amino acid sequence. In this case, the modified S6 (#20) construct was created via 10 nucleotide changes (Figure 5A) and RNA synthesized. The modified S6 RNA together with the 9 wt RNA segments were then introduced into BSR cells for virus recovery (15,18). After several attempts, virus could not be recovered, confirming the essential role of this region for viral replication (Figure 5B).

To determine whether virus recovery failed due to the failure of RNA packaging, which requires RNA–RNA interactions, we co-transcribed either the mutated S10 (#20) or mutated S6 (#20) together with the four (S6–S9 or S7–S10) wt segments. As predicted, the RNA shift mobility assay showed disruption of RNA complex formation and disappearance of certain retarded RNA bands. For mutated S10, only band A and C were visible (Figure 5C, lane 7), while for mutated S6 band D, was no longer visible (Figure 5C, lane 8), emphasising that not only these complexes are formed sequentially, but also S10 plays a leading role for RNA complex formation. The data is consistent with our previous report (8).

### Evidence of specific RNA interaction sites within the S10 RNA segment

**Figure 6.**
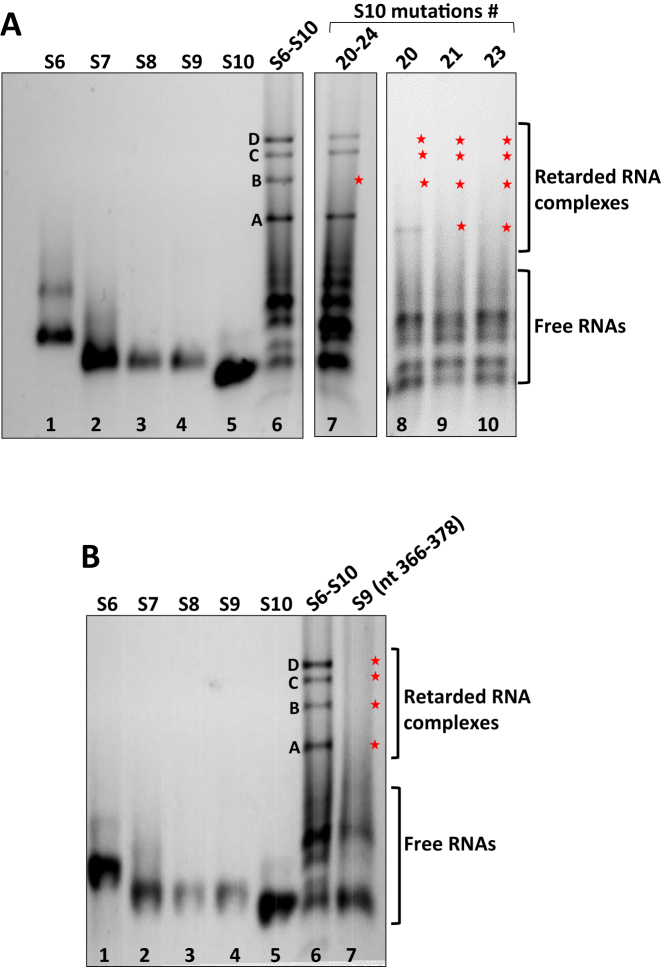
Visualisation of RNA Complex formation of the mutant S10 with other S6-S9 segments. (**A**) Native gels of complexes S6–S9 + S10 (#20–#24) (lane 7), S6–S9 + S10 (#20) (lane 8), S6–S9 + S10 (#21) (lane 9) and S6–S9 + S10 (#23) (lane 10). Retarded bands of complexes are indicated as A–D and absence of complexes shown in a star. Individual transcripts and co-transcription of wt S6–S10 complexes are used as controls. (**B**) Complex formation of the mutant S9 with S6–S8 + S10 wt (lane 7).

Several recent studies have revealed the essential function of the smallest RNA segment S10 in packaging and RNA–RNA interactions (8–11). Of the five predicted interaction sites, #20 of S6 appeared to be essential for formation of BTV RNA complex and virus replication. Therefore, we investigated further the importance of #20 and other interacting sites on S10. Three regions #20, #21 and #23, representing nts21–41, nts96–108 and nts447–460 of S10, respectively, were substituted with scrambled sequences (Supplementary Figure S5), followed by co-transcription with wt S6-S9 (Supplementary Figure S4), and EMSA analysis (Figure 6A). A complete inhibition of all RNA complexes (bands A–D) in mutations #21 (Figure 6A, lane 9) and #23 (Figure 6A, lane 10) was observed. The mutation at #20 also destroyed the retarded bands, except for the lower band A, which was still visible (Figure 6A, lane 8). Further, to examine the effect of mutation in all five predicted contact points (#20–#24) of S10 for RNA complex formation, multiple substitution mutations were introduced. Surprisingly, RNA shift gel showed that only the retarded band B RNA complex disappeared (Figure 6A, lane 7), while the remaining three bands, A, C and D were still present. These data suggested that RNA complexes are not only discrete but may be forming sequentially consistent with our previous data (8).

These *in vitro* mutagenesis data are also consistent with the predicted wt interaction network. Mutagenesis of #20 on S10 enables S6 to form a new contact with S9 (nts 366–378; –17.4kcal/mol), significantly changing the interaction network and thus accounting for the occurrence of a weak band A. In order to verify this, we also mutated this site on S9, co-transcribed it with other four wt segments and analysed it by native gel, which revealed the loss of all bands as expected (Figure 6B, lane7). The impact of the #20 region in S10 on virus replication was further tested by generating a mutant S10 genomic RNA, followed by virus recovery. There were only 10 nucleotide changes in S10 (Figure 5A), and no BTV was recovered using the mutant S10 (Figure 5B). These data confirm further that the predicted interaction sites are essential for virus replication.

**Figure 7.**
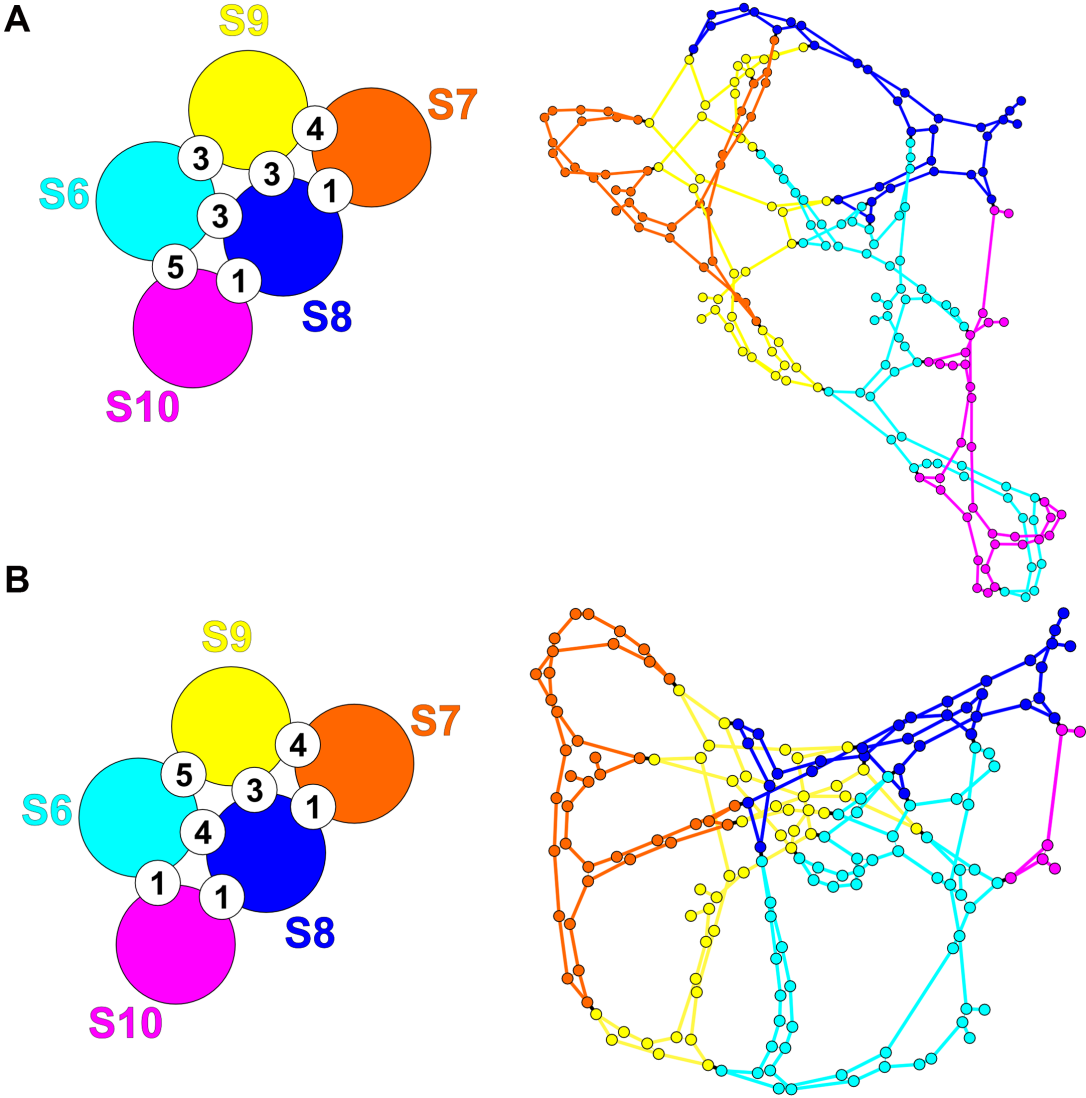
The predicted changes in interactions after mutagenesis of S10 sites. The numbers and topology of the interaction network for (**A**) S6–S9 + S10 (#23), and (**B**) S6–S9 + S10 (#20–#24) differ from those in wt virus (Figure 1).

Mutagenesis of #21 on S10 should not enable S6 to form any new compensatory contacts. Complex formation was however, completely ablated suggesting that this is an essential interaction, despite its relatively low interaction energy. Mutagenesis of #23 on S10 potentially enables a number of new contacts to form between S6 and S8 (nts 86–95, –15.6 kcal/mol; and nts 893–905, –17.5 kcal/mol) and S9 (nts 808–819, –17.8 kcal/mol), thus making it impossible to interpret experimental results based on the wt interaction network. We therefore recomputed the interaction network for this case, as well as for S6–S9 WT + S10 (#20–#24) (Figure 7). The dramatic changes seen in the position and linkage of the nodes in the network are consistent with the loss of all bands seen for the mutation at #23 (Figure 6A, lane10) whilst new contacts stabilising the network in Figure 7B are consistent with recovery of all but band B (lane 7).

### Importance of complementary sequences between S6 and S10 for complex formation

**Figure 8.**
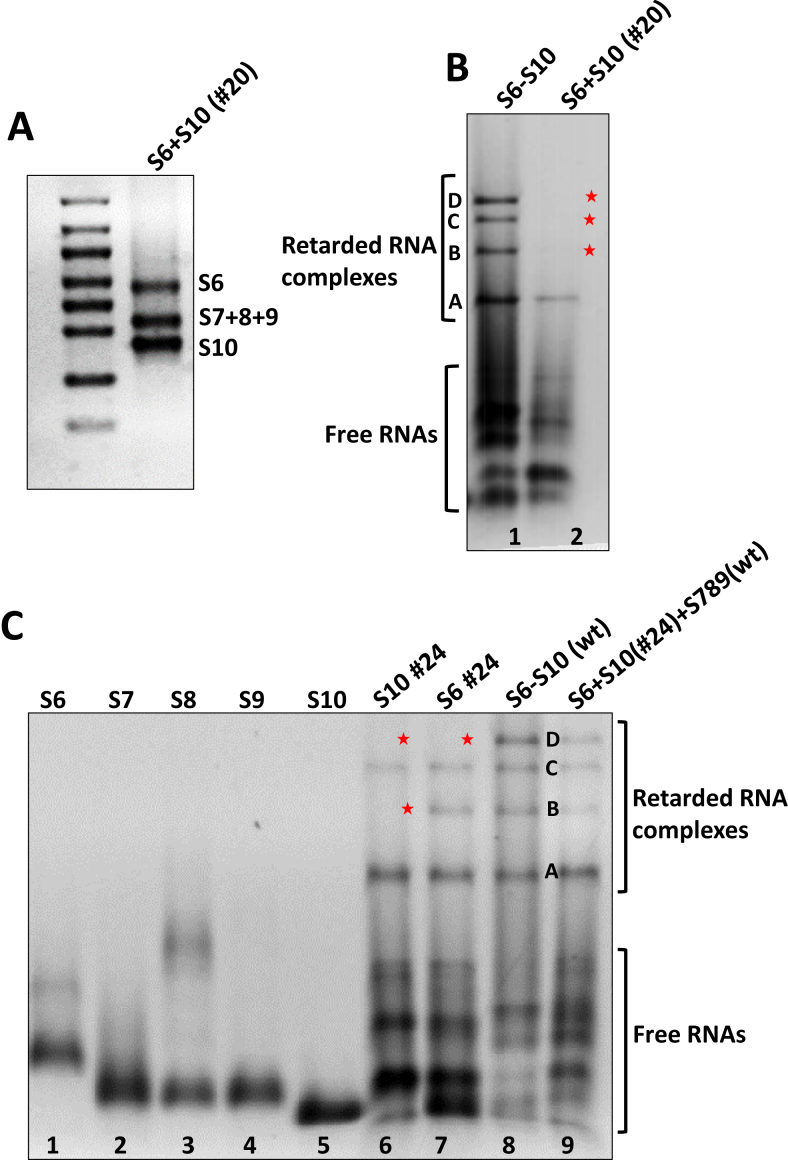
Effect of the complementary mutations on S6 and S10. (**A**) Co-transcription of segments S7–S9, together with S6 and S10 containing complementary mutations in the #20 region, is shown in a denaturing gel as five transcripts of correct sizes. (**B**) Native gel of complexes S7–S9 + S6 (#20) + S10 (#20) (lane 2) in comparison with wt control in lane 1. A star indicates the loss of RNA bands. (**C**) Native gel of complexes S6–S9 + S10 (#24) (lane 6), S7–S10 + S6 (#24) (lane 7) and S7–S9 + S6 (#24) + S10 (#24) (lane 9).

Of the five interaction sites between S6 and S10, the site #20 is the strongest by free energy. Mutagenesis in this region of S6 or S10 perturbed complex formation, confirming the importance of this contact. We investigated if it is possible to recover complex formation by introducing complementary mutations in S6 and S10 at this region. Both S6 and S10 were mutated at this site such that the sequences at #20 are complementary between these two segments, but differed from wt sequence (Supplementary Figure S5). Co-transcription reaction was then undertaken using these mutant S6 and S10 together with the wt S7–S9, followed by gel electrophoresis analysis. In native agarose gel, the signature complex bands, except for the band A, all other three bands were still absent (Figure 8B, lane 7), although all RNA transcripts were synthesized (shown in Figure 8A), indicating that this site cannot be complemented.

It is noteworthy that the complementarity between S6 and S10 in the #20 region was not sufficient to drive RNA complex formation, implying that not just the contact itself but also the sequence of specific nucleotides involved in this contact, are important. This is exactly what we expect from our analysis. As we have shown above, any mutations do not only affect the precise region mutated, but indeed the entire segment and its probabilities of forming *cis* or *trans* interactions anywhere on the segment. In particular, as discussed above, a trace of band A was visible after mutagenesis of S10, most likely due to a stabilizing effect on this band through a new bond formed between S6 and S9 that does not displace any other bond. Simultaneous mutation of #20 on S6 and S10 does not result in conflicting constraints and bond formation, and it is therefore not surprising that the same signature, i.e. band A, was seen following simultaneous mutagenesis of S6 and S10, thus providing an explanation based on the complex interaction network for why recovery was not possible.

Since it was not possible to compensate for the mutation at the #20 interacting site, we selected a weaker interaction site between these two RNA segments. Mutation at #24 on S6 could still generate three of the four signature bands (Figure 3B, lane 8), indicating the weaker effect of this site. Therefore, we tested if this site could be compensated for by an appropriate S10 mutation at the same site leading to formation of all four complexes in the presence of this S10 mutation, and we generated the S10 mutation accordingly (Supplementary Figure S5). The mutated S10 was then co-transcribed with the other four wt segments, or both S10 and S6 mutated segments with the S7–S9 wt segments. As shown by native gel, the RNA complexes were perturbed when the S10 mutant was used in isolation (Figure 8C, lane 6), but in the presence of the mutant S6, all four bands were recovered, confirming the complementarity between the two mutant segments (Figure 8C, lane 9).

## DISCUSSION

A key process in successful virus infection is the sorting of the viral genome from other nucleic acids in the virus-infected cytosol for packaging into the assembling particle. This is particularly so for viruses with segmented genomes such as Influenza virus, Rotavirus and BTV. The difficulty in understanding these interactions is confounded by the plethora of different secondary structures that a genomic RNA may assume, especially when interacting with other RNA molecules (8,9,19–22, BioRxiv: https://doi.org/10.1101/236620). Here, we have introduced a bespoke network approach to predict the possible RNA–RNA interactions between different segments, and have provided proof-of-principle of its predictive power using molecular assays and virus recovery. In particular, we identified vital *trans* interaction sites in the formation of RNA complexes among the five smaller segments of BTV, i.e. S6–S10. Data from *in vitro* RNA–RNA interaction assays, substantiated by isolating the assembled five segments as one single band via a sucrose gradient ultracentrifugation as in Lourenco and Roy (16), validate the theoretical model (see Figure 1). Further, four different bands visualised by gel electrophoresis all contain five segments, despite the variable ratios of different segments in each. It is possible that the band C, with the equal ratio of all segments corresponds to an RNA complex that preferentially recruits the remaining segments prior to being assembled in the viral capsid.

Mutations of the predicted RNA–RNA interaction sites—and indeed even minor changes to one or a small number of the five predicted contacts between S6 and S10—were found to be attenuating or lethal when rescued into live virus, consistent with their predicted role(s) in the complex. Indeed, the major consequences of such mutations are expected from the model, because these mutations have dramatic consequences for the structure and topology of the interaction network (as e.g. in Figure 7A) because of the complex interdependencies of different interaction probabilities in the interaction network. Interestingly, changes to an S6–S10 contact can have distinct effects on complex formation and network topology depending on whether the mutation is introduced on the S6 or on the S10 site of the contact. Mutations carried out at one site can affect other, even distant, sites in the network (as we have demonstrated for #20, the strongest predicted interaction site). Mutation #24 of S6 or S10 had a lesser effect on complex formation, because they have had a less dramatic impact on the topology of the interaction network. This demonstrates that our network approach can be used to identify sites with the strongest impact on complex formation, which could be useful when identifying potential drug targets.

As discussed above, the only band out of the four, band C, is the correct complex for further genome packaging, and other three bands might represent different stages in the sequential complex formation in different conformations. Alternatively, these complexes might represent conformations, in which alternative, transient contacts are made between the segments that are later replaced by those characteristic of the final complex. However, how this preferred complex is selectively packaged is yet to be revealed. Recent data suggest that in the absence of VP6, i.e. the RNA binding helicase protein of BTV, the RNA complex fails to be packaged into the assembling capsid. Further, the interaction of capsid proteins VP3 and VP6 is essential for genomic RNA recruitment (23,24). These data suggest a crucial role for protein-RNA interaction during assembly of the RNA complex, which is probably necessary for large, segmented RNA viruses. In particular, the non-structural protein NSP2 in rotavirus binds to the ssRNA segments, triggering conformational changes in their secondary structures that are conductive to the formation of stable trans interactions (25). Such effects can be included in our network approach. For example, if the binding sites of the NSP2 proteins on the RNA are known, e.g. via CLIP-Seq, then the RNA segments corresponding to these areas can be forced to be non-interactive with RNA, and RNA–RNA contacts (both cis and trans) be identified conditional to that. The methods introduced in this paper are therefore of wider relevance, and applications include those more complex viral systems.

It had previously been demonstrated based on *in vitro* assembly assays and protein-free RNA–RNA interaction assays that genomic RNA segments recognise each other (8), but the nature of these contacts and the topology of the complex interaction network have remained an open problem that could not be resolved with experiment alone. Here, we have been able to predict these interactions for the first time and study the topology of the interaction network, thus identifying *trans* interactions that are crucial for segment assortment and formation of an RNA complex for packaging into the protein container. The RNA sites involved in key *trans* interactions are reminiscent of, yet distinct from, the packaging signals discovered recently in different viral families (26–28). They could be important for both icosahedral viruses, such as BTV, and non-icosahedral viruses, such as what is shown for influenza virus (19,21,22,29).

Taken together, the network approach introduced here can identify a number of inter segment contacts crucial for segment assortment and RNA complex formation, a prerequisite for packaging. We expect that this method, in concert with experiment, will enable new avenues for anti-viral intervention against viruses that either inhibit or misdirect RNA segment assortment.

## Supplementary Material

Supplementary Data
