# Supplementary material for: Dynamic network approach for the modelling of genomic sub-complexes in multi-segmented viruses
Source: Nucleic Acids Res. 2018 Oct 9;46(22):12087–98. doi: 10.1093/nar/gky881 (PMC6294558; doi:10.1093/nar/gky881)

## Slide 1
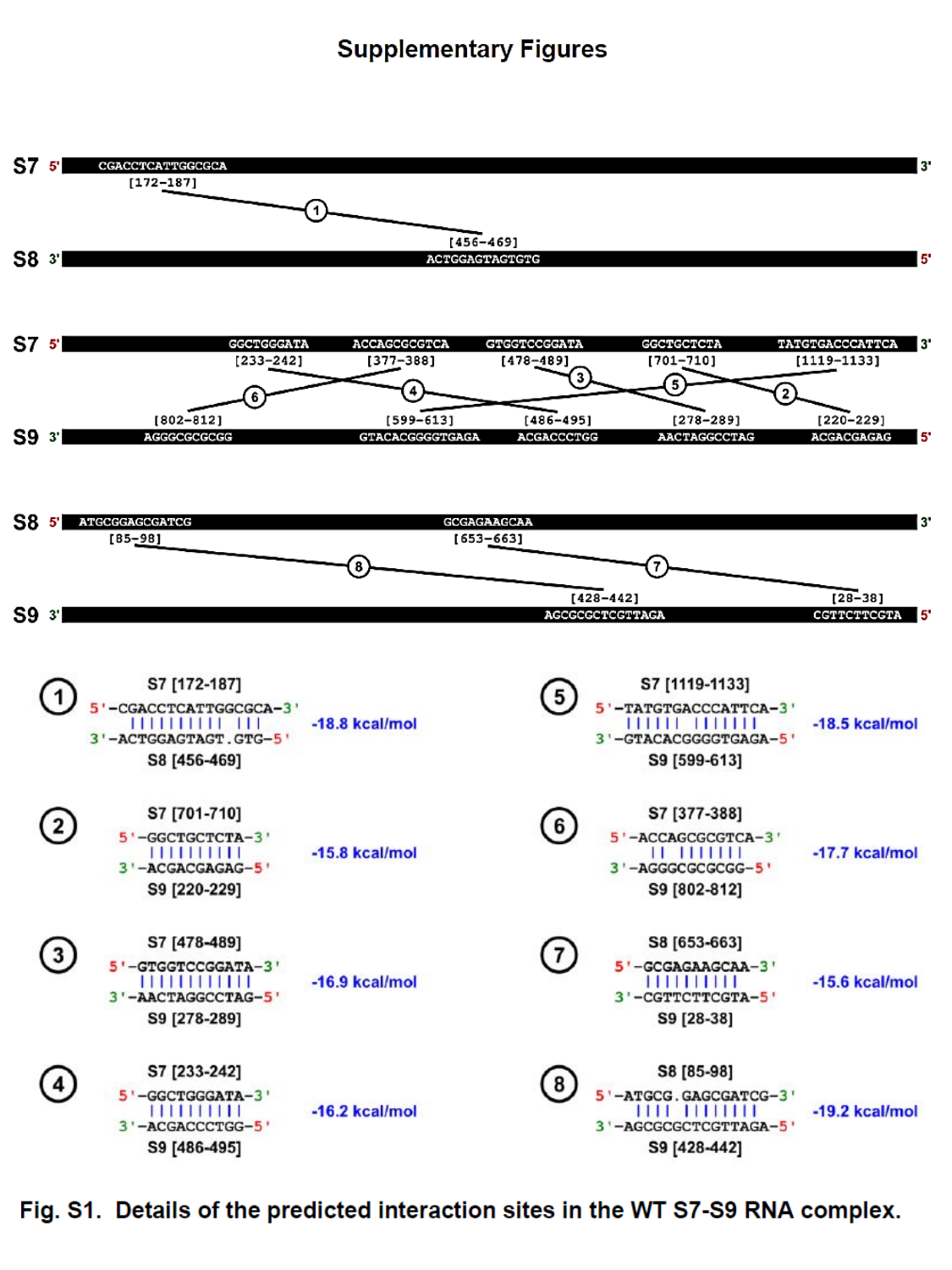

## Slide 2
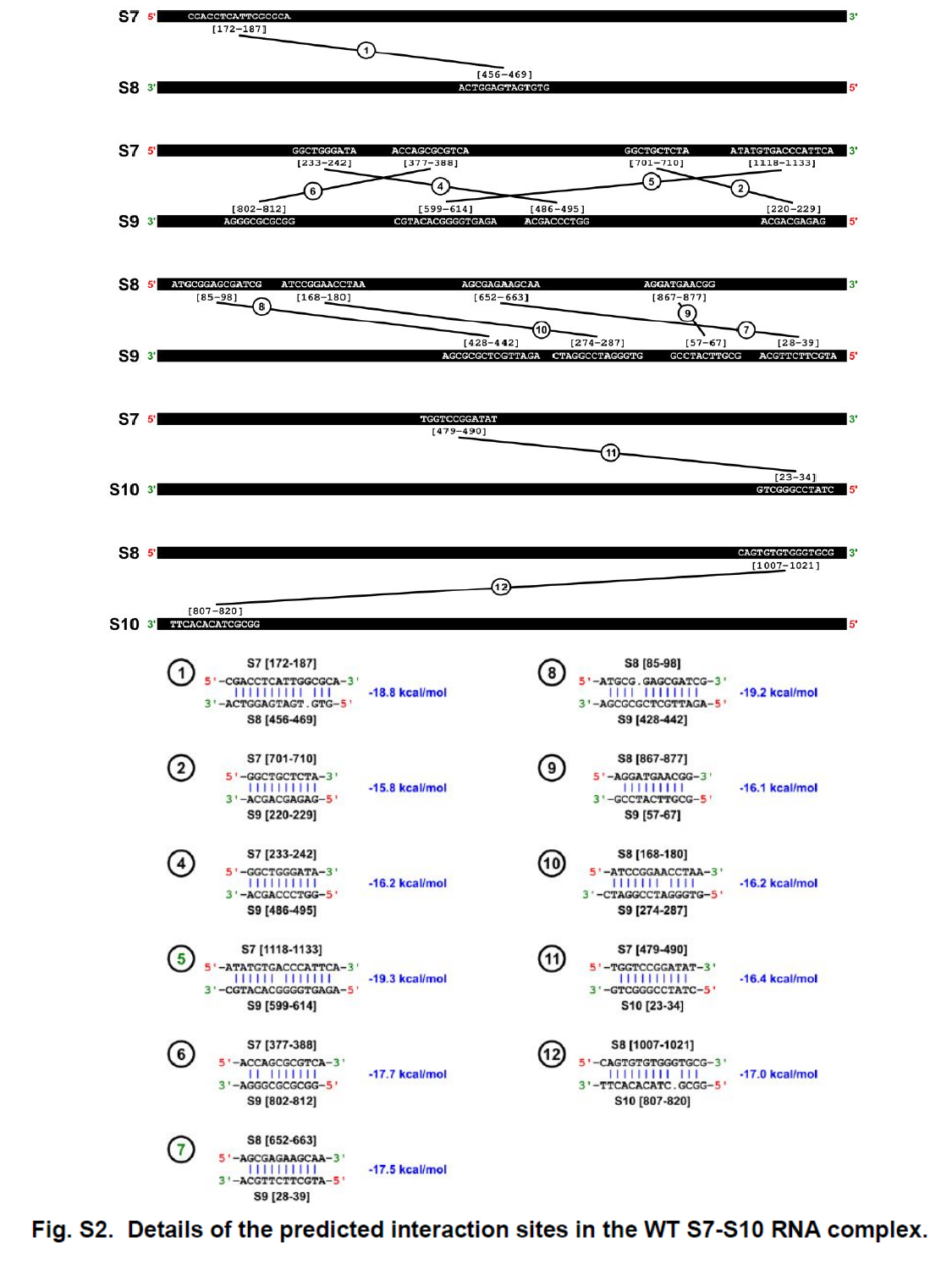

## Slide 3
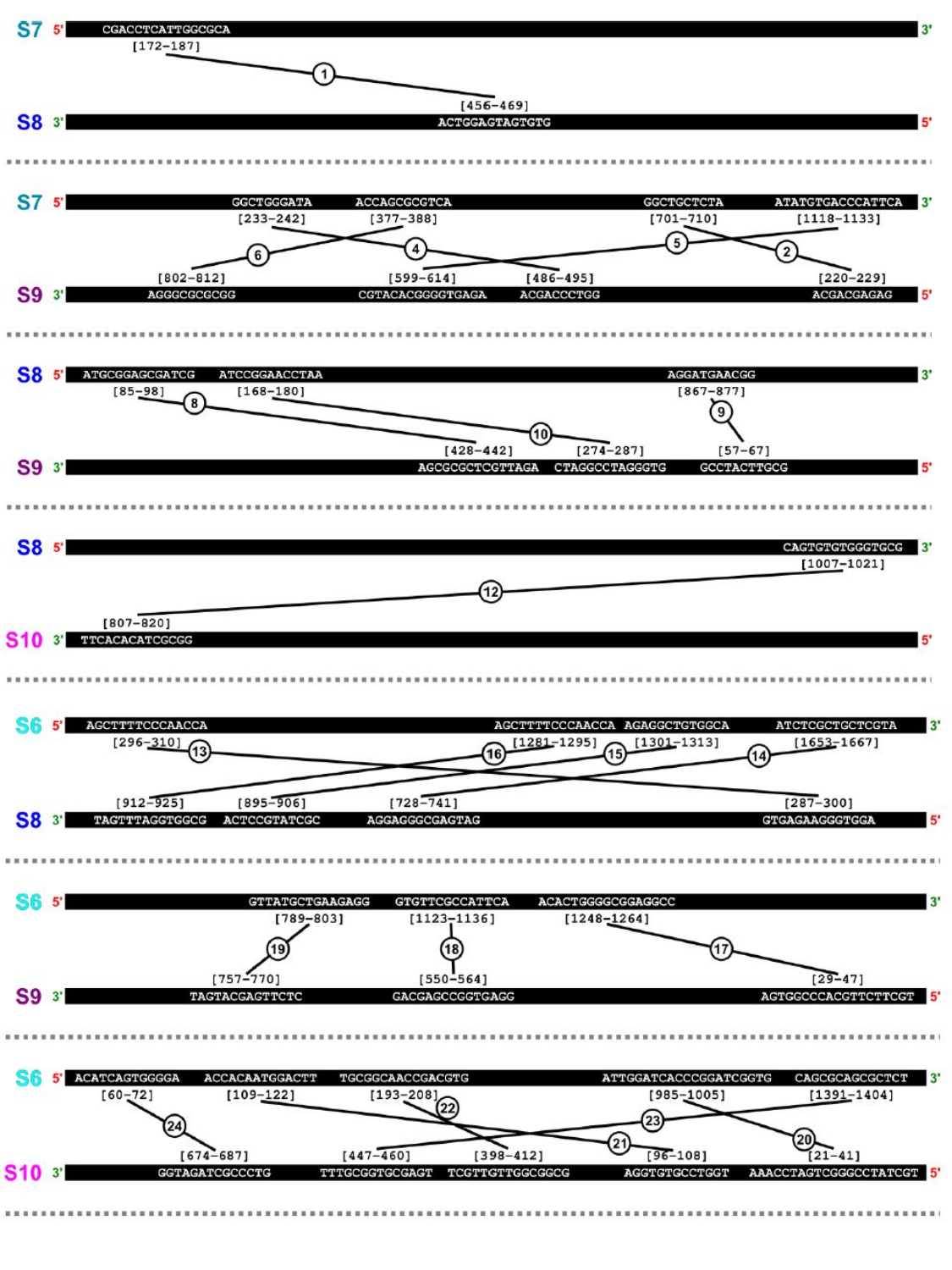

## Slide 4
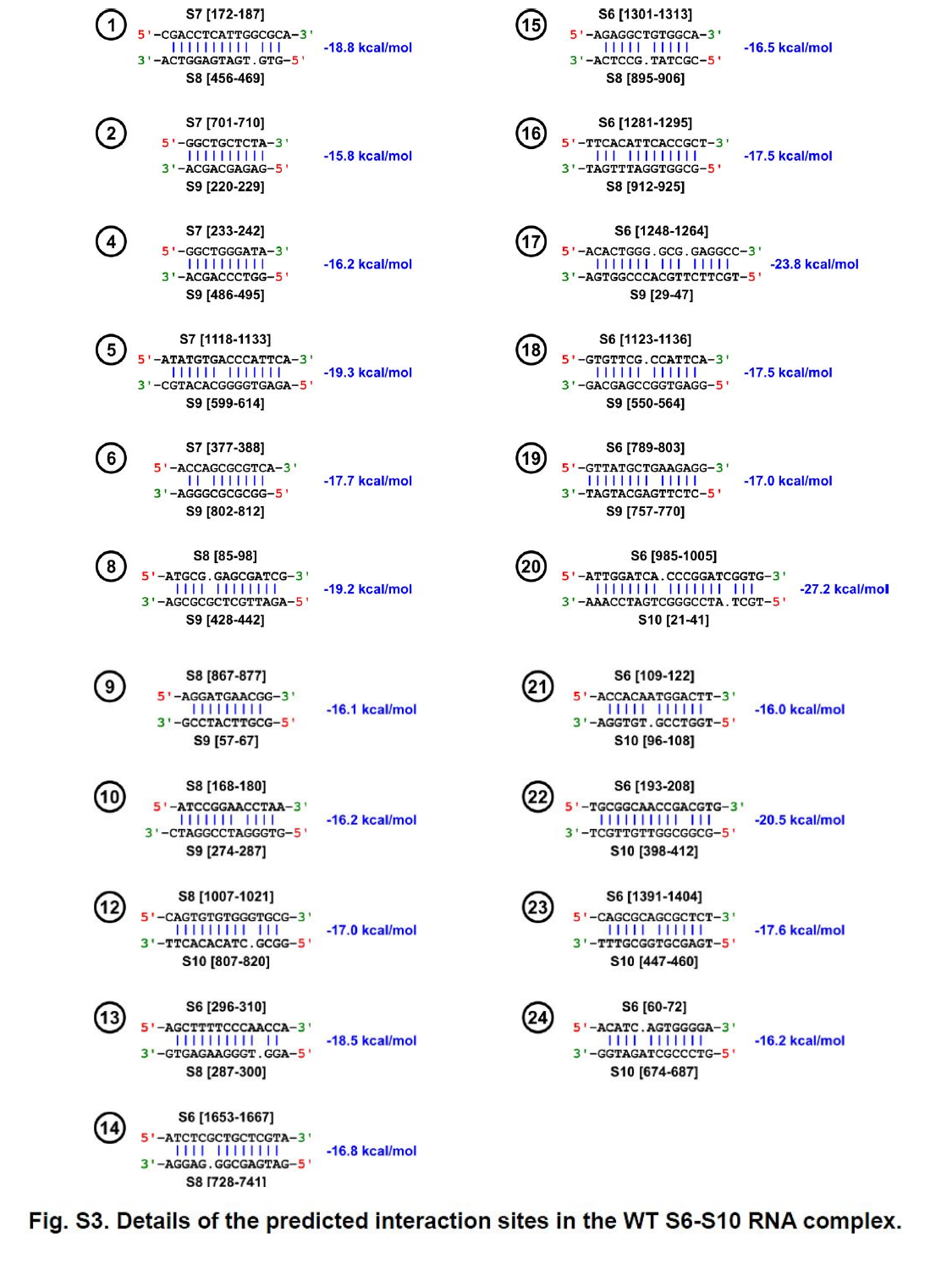

## Slide 5
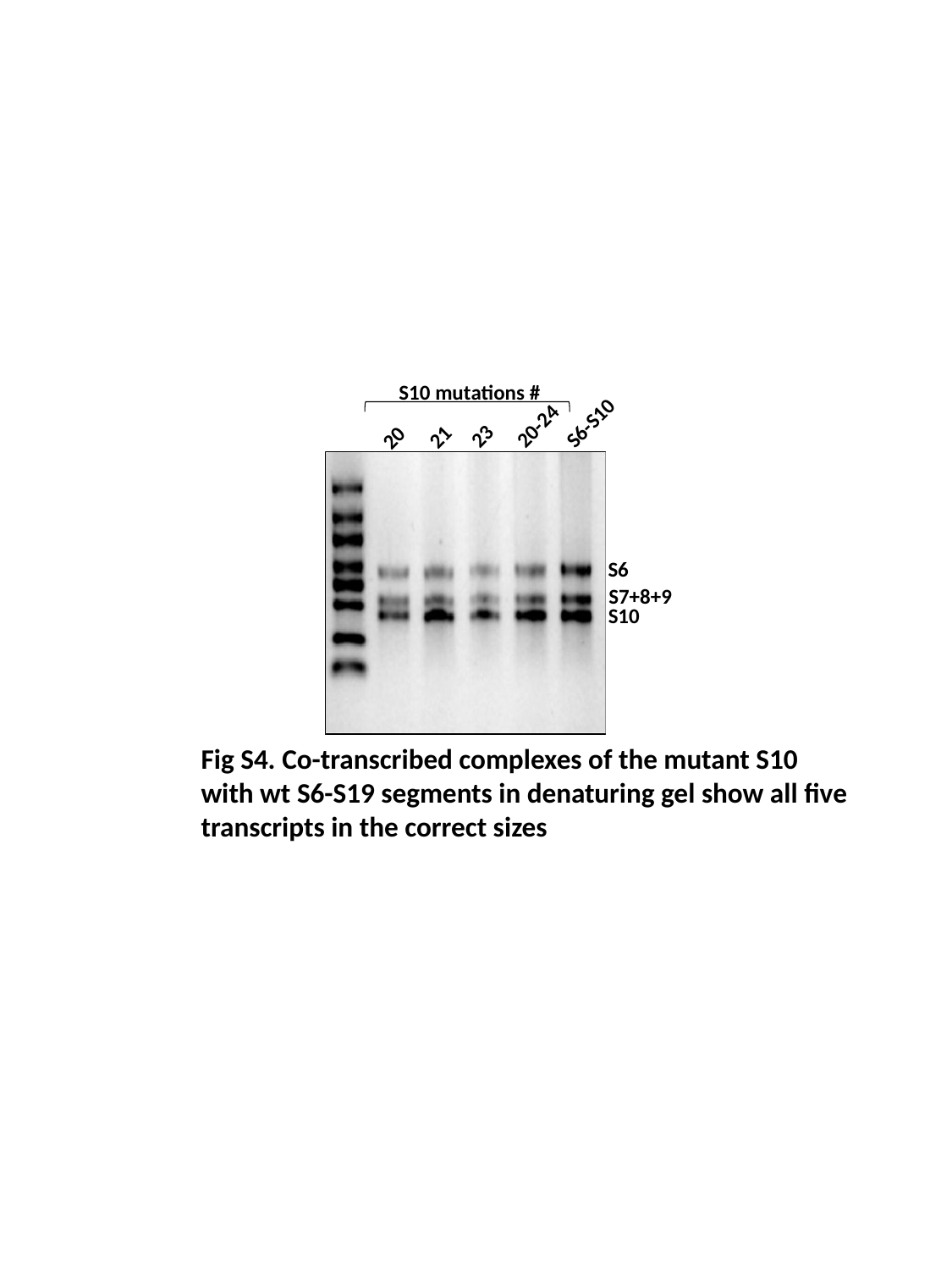

S10 mutations #
S6-S10
20-24
21
23
20
 S6
 S7+8+9
 S10
Fig S4. Co-transcribed complexes of the mutant S10 with wt S6-S19 segments in denaturing gel show all five transcripts in the correct sizes

## Slide 6
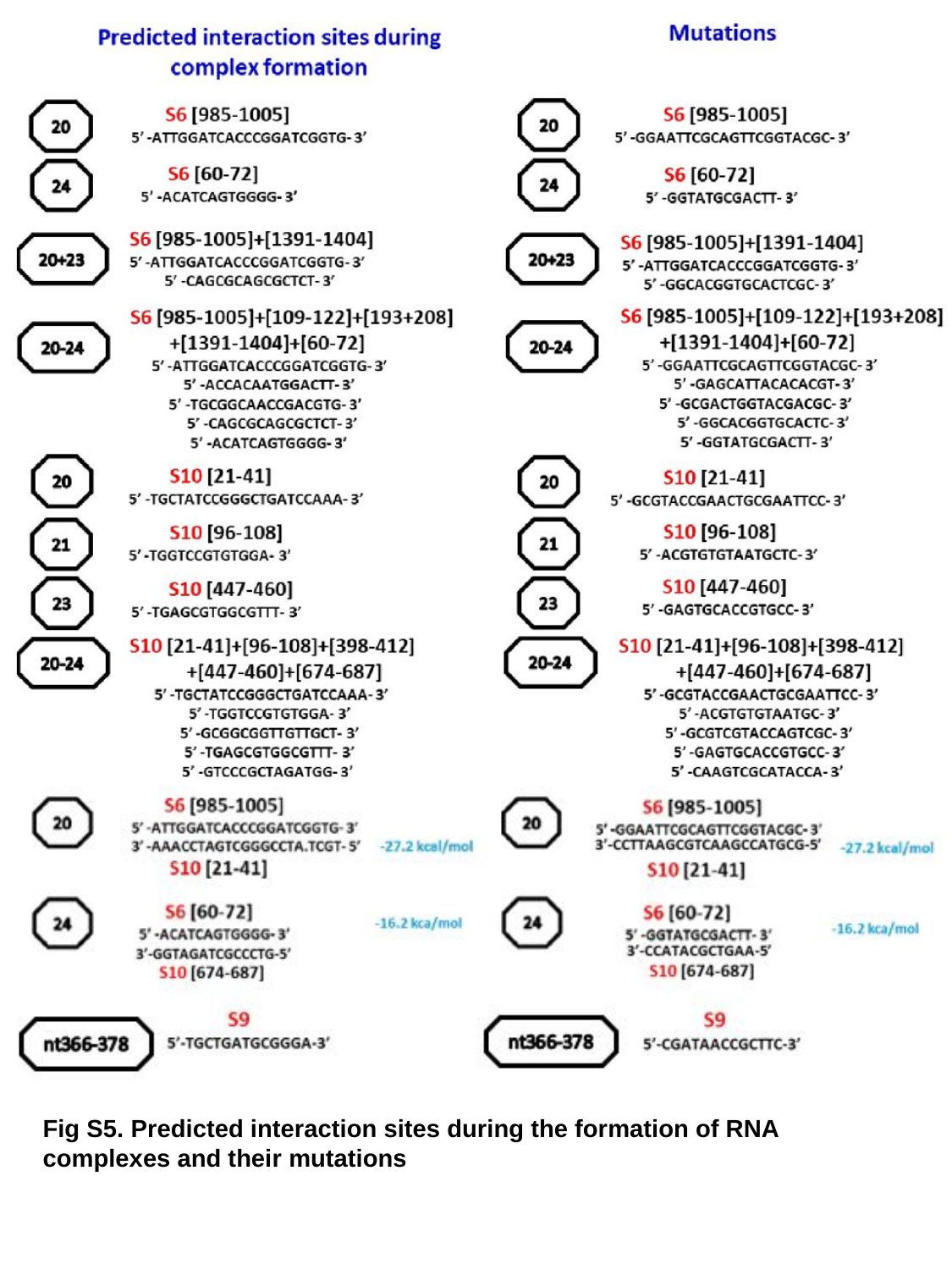

Fig S5. Predicted interaction sites during the formation of RNA complexes and their mutations

## Slide 7
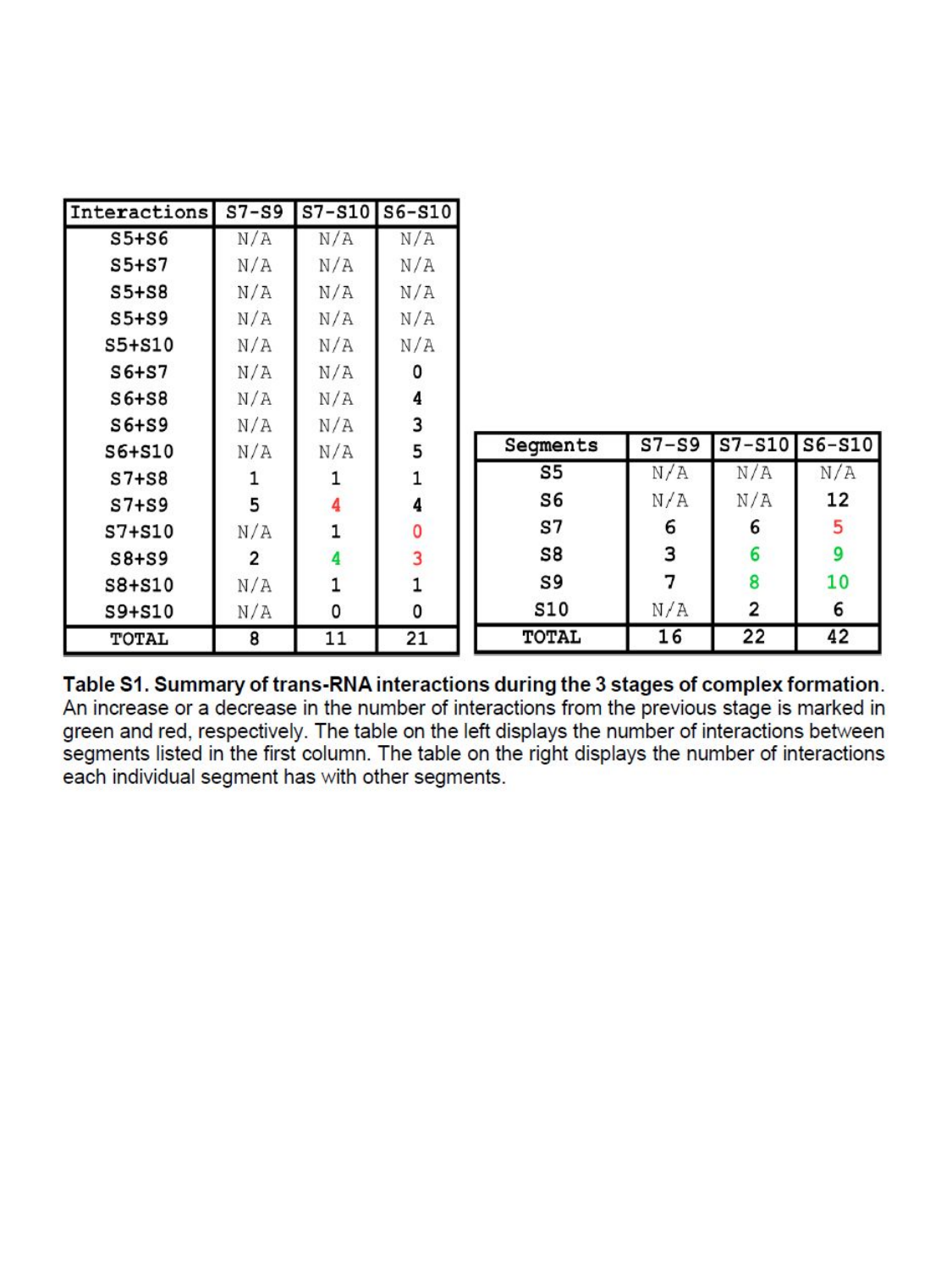

## Slide 8
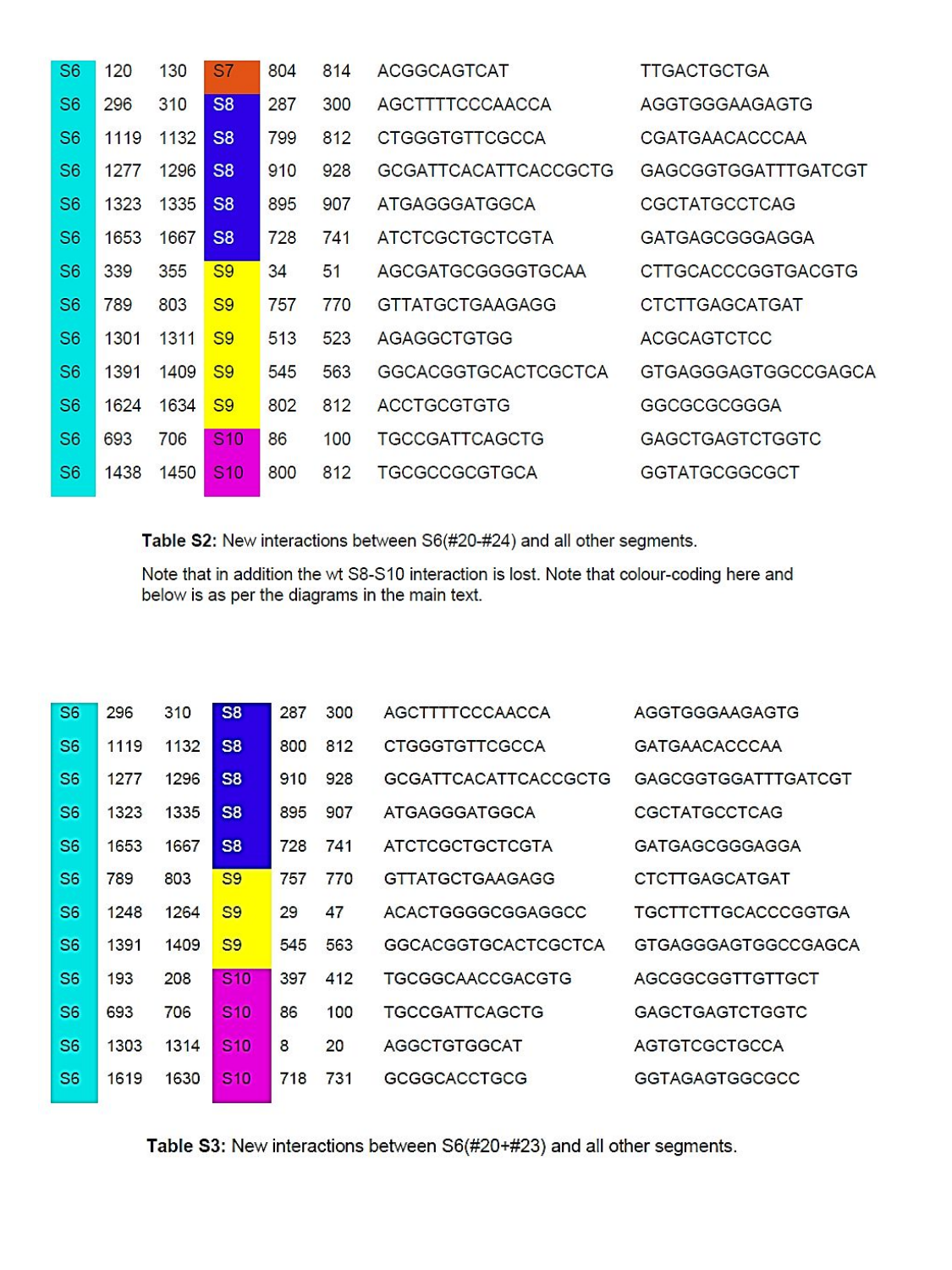

## Slide 9
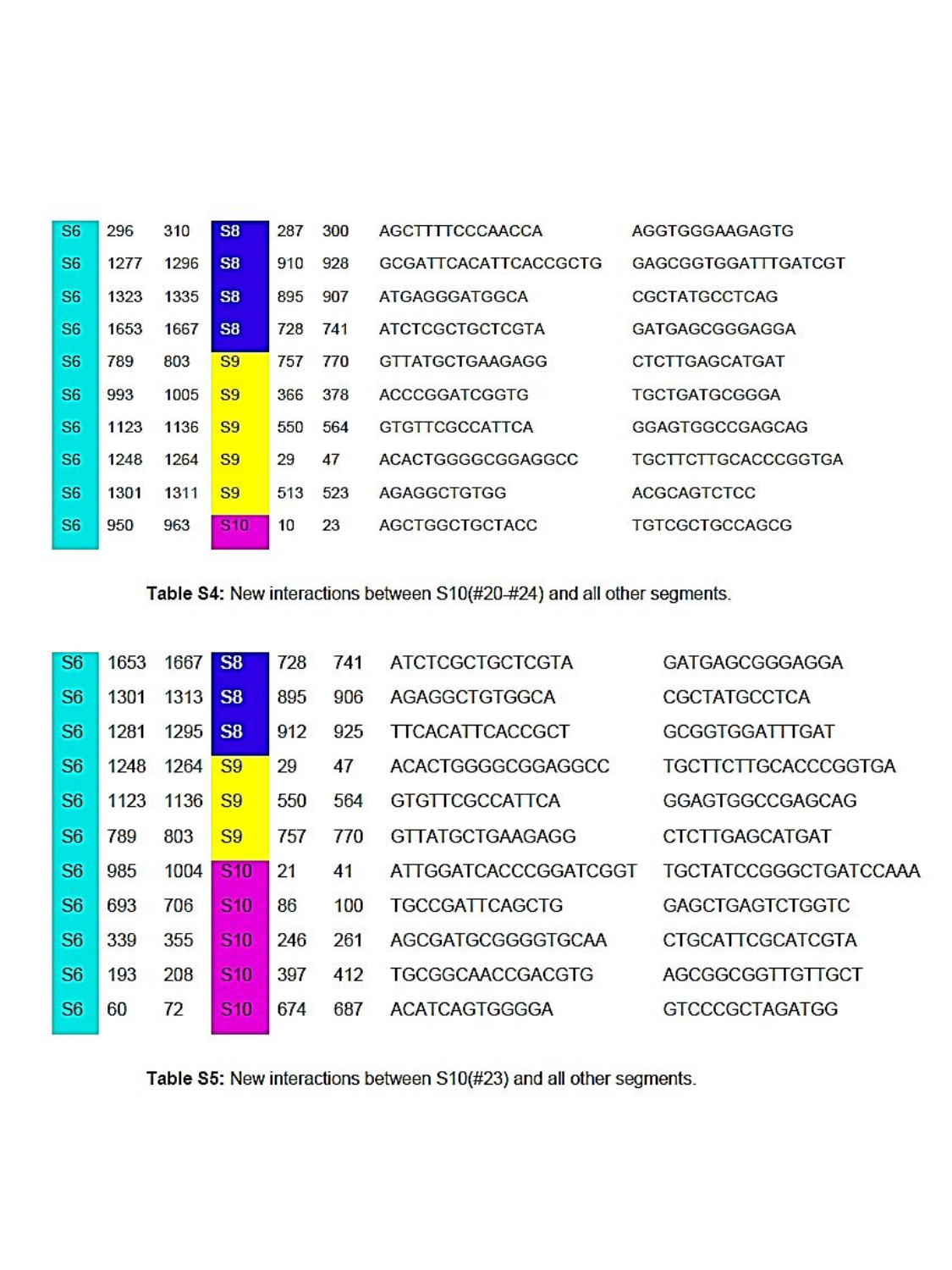

Supplement: Supplementary Data [file gky881_supplemental_files.pptx]
